# Growth differentiation factor‐15 and the risk of cardiovascular diseases and all‐cause mortality: A meta‐analysis of prospective studies

**DOI:** 10.1002/clc.23159

**Published:** 2019-03-26

**Authors:** Shanhui Xie, Liping Lu, Liwei Liu

**Affiliations:** ^1^ Department of Clinical Laboratory Shengjing Hospital of China Medical University Shenyang China

**Keywords:** all‐cause mortality, cardiovascular diseases, growth differentiation factor‐15, meta‐analysis

## Abstract

**Background and Aim:**

Previous studies have documented that the association between growth differentiation factor‐15 (GDF‐15) the risk of patients with cardiovascular diseases (CVDs). In this meta‐analysis, our main objective is to explore the associations between GDF‐15 and the risk of CVD or all‐cause mortality.

**Methods:**

PubMed and ISI Web of Science (up to January 2018) electronic databases were browsed for eligible studies. The studies provided relevant data depicted as hazard ratio (HR) with 95% confidence interval (CI), with regard to the association between GDF‐15 levels and subsequent risk of CVDs or all‐cause mortality. A random‐effect model was applied to pool the HR and 95% CI.

**Results:**

Thirty‐one prospective studies met the eligibility criteria involving 53 706 subjects with 7020 adverse outcome events. It was concluded that GDF‐15 levels were associated with an incremental risk of CVDs or all‐cause mortality. Highest GDF‐15 category was associated with greater risk of cardiovascular mortality (HR, 2.66; 95% CI, 1.69‐3.63), all‐cause mortality (HR, 2.52; 95% CI, 2.06‐2.97), and complex adverse outcome (HR, 1.81; 95% CI, 1.42‐2.21). As each log‐unit increment in GDF‐15 concentration, the corresponding risk of adverse events also escalated, cardiovascular mortality (HR, 2.11; 95% CI, 1.57‐2.66), all‐cause mortality (HR, 2.70; 95% CI, 2.29‐3.12), and complex adverse outcome (HR, 1.96; 95% CI, 1.64‐2.29).

**Conclusions:**

Judging from the results of the data analysis, GDF‐15 levels may increase the risk of CVDs or all‐cause mortality.

## 1 INTRODUCTION

Growth differentiation factor‐15 (GDF‐15), first named as macrophage inhibitory cytokine‐1 (MIC‐1),was a stress‐response member of transforming growth factor‐β cytokine superfamily. It was found that GDF‐15 messenger RNA (mRNA) expressing increased during macrophage activation.^1^ Normally GDF‐15 is weakly expressed in most tissues under physiological conditions but its expression level may sharply upregulate in response to ischemia‐reperfusion injury, reactive oxygen species, and mechanical stretch, possibly mediated through pro‐inflammatory cytokine and oxidative stress dependent signaling pathways.^2^, ^3^ Moreover, it had suggested that elevated GDF‐15 was a cardioprotective cytokine when exposed to cardiovascular injury in an animal model.^3^ In humans, increased GDF‐15 had been observed in atherosclerotic plaque macrophages.^4^

To date, cardiovascular diseases (CVDs), a leading cause of mortality worldwide, have brought heavy burden to social healthcare and individuals. Thus, intensive investigation has been focused on controlling the risk factors aimed at lowering CVD risks. Plenty of clinical research has been conducted to explore the relationship between GDF‐15 levels and CVDs. These experiments conclusively demonstrate that GDF‐15 levels link to the adverse cardiovascular events across a spectrum of CVD conditions including heart failure (HF), chest pain, acute coronary syndromes (ACS), stable ischemic heart disease, stroke, and atrial fibrillation.^5^, ^6^, ^7^, ^8^ The potential ability of GDF‐15 may attribute to the earlier diagnosis, risk stratification and prognosis assessment. However, no systematic review and meta‐analysis have analyzed the available data pertaining to the association between GDF‐15 levels and CVDs or all‐cause mortality. Hence, we perform a meta‐analysis for the purpose of qualitatively and quantitatively assessing the relationship between GDF‐15 levels and CVDs or all‐cause mortality.

## 2 MATERIALS AND METHODS

### 2.1 Literature Search

Following the Preferred Reporting Items for Systematic Reviews and Meta‐Analysis guideline, we searched PubMed and ISI Web of Science databases from January 1, 1950 to December 31, 2017 for the terms “growth differentiation factor‐15” or “macrophage inhibitory cytokine 1”or “placental transforming growth factor beta” or “non‐steroidal anti‐inflammatory drug‐activated gene‐1” or “prostate‐derived factor” and “cardiovascular disease” or “coronary heart disease” or “ischemic heart disease” or “myocardial infarction” or “heart failure” or “stroke” or “all‐cause mortality”or “acute coronary syndrome” or “troponin.” The retrieval process was independently completed by two authors (S. Xie and L. Liu). In addition, we also retrieved the reference list of the selected studies and recent reviews for obtaining further information. The literature search was restricted to human studies and published in English language.

### 2.2 Inclusion and exclusion criteria

Studies were eligible for inclusion if they met the following criteria: (a) prospective cohort study, (b) follow‐up duration of at least 3 months, (c) reported at least one of the interesting outcomes: major cardiovascular endpoints (cardiovascular death, myocardial infarction, stroke, and HF) or all‐cause mortality, (d) provided relative risk (RR) or hazard ratio (HR) with 95% confidence interval (CI) for GDF‐15 levels comparing the highest levels to the lowest or definite increases for risk factor as a continuous variable (data after logarithmic transformation). studies were excluded if the study design was review articles, case‐controlled studies ,retrospective cohort studies, commentaries, editorials, or case report; studies concerning ecological ,animal, or cell culture, genetic variation in GDF‐15 relevant genes were not selected; we also excluded the study population based on non‐adult.

#### 2.2.1 Data extraction

Two investigators (S. Xie and L. Liu) performed the relevant data extraction with discrepancies reconciled through deliberation with a third investigator (L. Lu).The information was extracted as follows: authors, publication year, sample size, characteristics of the baseline population, mean age of the participants, study location, mean levels of GDF‐15, detective method, follow‐up, study endpoints, total number of related events, RR or HR with 95% CI, covariates adjusted for in multivariable analyses. The data from different sub‐cohort of the same study was extracted separately.

### 2.3 Assessment of study quality

According to the Newcastle‐Ottawa quality assessment scale (for cohort study), the assessment of including study quality was performed following three aspects: participants selection, comparability of groups, and ascertainment of outcome. A study can be awarded a maximum of one score for each numbered item within the selection. A maximum of two scores can be given for comparability and selection. Higher scores of studies represented better quality.

### 2.4 Statistical analyses

Various studies reported the results in different patterns and presented the effect sizes for comparison between groups or for a given unit of increase in GDF‐15 levels. To display the results more concise and understandable, we assessed the association between GDF‐15 categorical level and the risk of CVD mortality or all‐cause mortality or complex adverse outcome (composite of death or cardiovascular events), by comparing the highest category of GDF‐15 with the lowest. We also analyzed GDF‐15 level as a continuous variable to evaluate the risk of CVD mortality or all‐cause mortality or complex adverse outcome based on per log‐unit increase of GDF‐15. Pooled HR with 95% CI was presented as an effect size for estimating the association between GDF‐15 levels and the risk of CVD mortality or all‐cause mortality or complex adverse outcome. In the process of handling the data, the SD of log‐GDF‐15 was estimated from the Framingham Heart Study, Framingham, MA.^9^ A random‐effect model was applied to pool the data across studies.^10^ For assessing the extent of divergence, the heterogeneity of trials was examined by Q‐statistic and quantified by the I^2^ statistic. It was considered that a relatively larger extent of I^2^ represented greater heterogeneity. We undertook meta‐regression analysis to explore the possible reason resulted in heterogeneity. According to the different characteristics of including studies, subgroup analyses were performed, respectively, stratified by the sample size (≤1000 or >1000), duration of follow‐up (≤5 years or>5 years), assay method (enzyme‐linked immunosorbent assay, radioimmunoassay, or others), whether to adjust the variable, and baseline population (general population, coronary heart disease, others). Sensitivity analysis was performed to appraise an excessive estimate of a single study by way of eliminating each study individually. An estimation of potential publication bias was performed by both Egger's linear regression test. All statistical analysis was performed with software package Stata version 12.0 (STATA Corp LP, Texas). *P* < 0.05 was identified with a statistical significance.

## 3 RESULTS

### 3.1 Identification of studies

**Figure 1:**
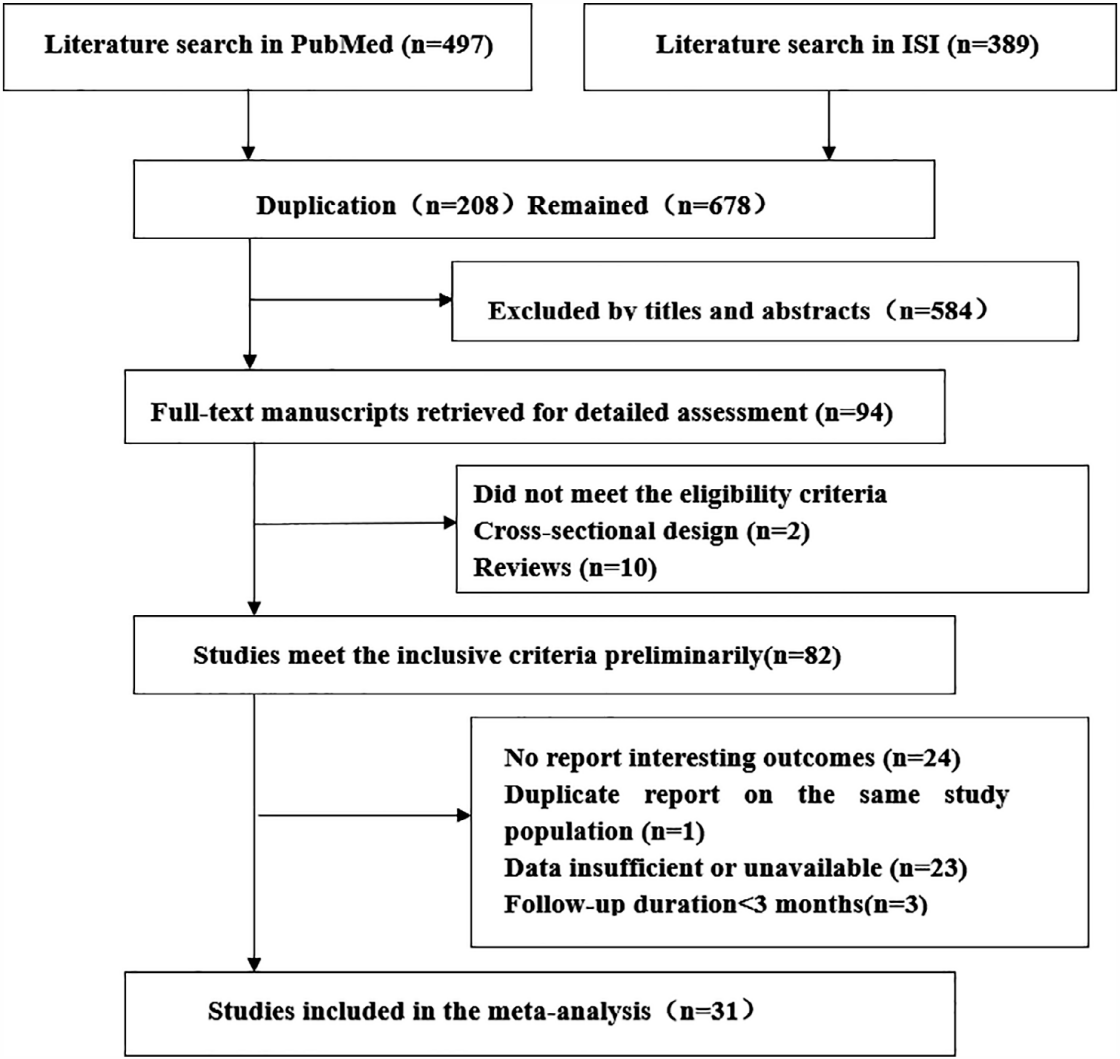
Flow chart of study selection process for meta‐analysis

The procedures of literature retrieval and selection were present in Figure 1. We initially retrieved 886 relevant publications from the PubMed and ISI Web of Science electronic databases. The majority of these were excluded after screening the titles or abstracts, because of editorials/reviews/case reports/cross‐sectional design or not related. Twenty studies did not provide interesting outcomes, 18 articles were excluded because the data provided were insufficient or unavailable, one study was excluded because of duplicate report on the same study population, and one study follow‐up duration was less than 3 months. Finally, 29 studies were selected in our meta‐analysis.

### 3.2 Study characteristics

Table 1 displayed the baseline characteristics of participants across the 29 studies. The eligible trials involved a total of 53 706 participants. Six^9^, ^20^, ^21^, ^22^, ^23^, ^28^ studies enrolled community‐based populations, 17^6^, ^11^, ^12^, ^13^, ^14^, ^15^, ^16^, ^17^, ^18^, ^19^, ^27^, ^29^, ^31^, ^32^, ^34^, ^36^, ^37^ studies were restricted to patients with coronary artery disease (CAD), five^5^, ^7^, ^24^, ^25^, ^30^ studies were on HF, and the remaining three were respectively pertaining to diabetes mellitus (DM),^26^ atrial fibrillation (AF)^33^ and intensive care unit patients.^35^ The mean age of the subjects ranged from 42 to 79 years. The median follow‐up duration ranged from 0.25 to 11.3 years. Table 2 showed the estimates of association between GDF‐15 levels and risk of adverse outcomes included in the meta‐analysis. Nineteen^5^, ^7^, ^11^, ^12^, ^13^, ^14^, ^15^, ^16^, ^18^, ^21^, ^23^, ^24^, ^25^, ^27^, ^29^, ^30^, ^34^, ^36^, ^37^studies individually regarded the GDF‐15 level as a continuous variable to evaluate the risk of CVDs, 7^6^, ^17^, ^20^, ^26^, ^28^, ^32^, ^33^ studies only regarded the GDF‐15 level as a categorical variable and 5^9^, ^19^, ^22^, ^31^, ^35^ studies utilized both two ways. Among the 31 trials, seven reported CVD mortality as outcomes, 13 reported complex adverse outcome, and 20 reported all‐cause mortality as outcomes. And the estimate of association between GDF‐15 levels and risk of adverse outcomes of included studies was yielded in the Table 2.

**Table 1:** Baseline characteristics of the identified studies

Source	No. of Patients	Baseline population	Nation	Age (y)	Follow‐up (y)	Events number	Assay Method	Quality score
Wollert et al,^11^	2079	NSTE‐ACS(GUSTO‐IV trial)	Germany	66	1	143	RIA	7
Eitel et al,^12^	238	STEMI (LIPSIA‐N‐ACC)	Germany	67	0.5	36	ELISA	6
Kempf et al,^13^	741	AMI	Germany	67	1	59	RIA	6
Eggers et al,^14^	950	NSTE‐ACS (FRISC II trial)	Sweden	67.1	5	220	RIA	7
Kempf et al,^15^	2229	CHD (AtheroGene Study)	Germany	61.5	3.6	188	RIA	7
Khan et al,^16^	1142	AMI	UK	67	1.38	303	ELISA	6
Damman et al,^17^	1151	NSTE‐ACS(ICTUS)	The Netherlands	62	5	236	RIA	7
Lin et al,^18^	216	STEMI	Taiwan	59.8	2.33	18	ELISA	7
Schopfer et al,^6^	984	Ischemic heart disease	USA	66.7	8.9	478	ELISA	7
Bonaca et al,^19^	3501	ACS (PROVE IT‐TIMI 22 trial)	USA	58.1	2	317	RIA	7
Izumiya et al, 2014^7^	149	Heart Failure	Japan	69.9	1.96	16	ELISA	7
Wang et al,^9^	3428	General population (FHS)	USA	59	11.3	824	ECLIA	9
Daniels et al,^20^	1740	General population (RBS)	USA	71	11	521	ELISA	8
Eggers et al,^21^	1004	General population (PIVUS)	Sweden	70	8	111	RIA	8
Rohatgi et al,^22^	3291	General population (DHS)	USA	48.7	7.3	120	ELISA	9
Wallentin et al,^23^	940	General population (ULSAM)	Sweden	71	9.8	265	ECLIA	9
Lok et al,^24^	209	DEAL‐HF	The Netherlands	71	8.7	151	ECLIA	8
Foley et al, 2009^25^	158	Patients with heart failure undergoing CRT	UK	68	2.6	52	ELISA	7
Lajer et al,^26^	891	Type 1 diabetic patients With Nephropathy	Denmark	42.1	8.1	229	ECLIA	9
Schnabel et al,^27^	1781	CAD (AtheroGene Study)	Germany	63	3.6	137	ELISA	7
Wiklund et al,^28^	876	Male cohort (general population)	Sweden	68	5.3	102	ELISA	7
	324	Twin cohort (general population)		78.6	9.1	214		
Kempf et al, 5	455	Chronic Heart Failure	Germany	64	3.33	117	RIA	6
Widera et al,^29^	754	NSTE‐ACS	Germany	70	0.5	66	RIA	6
Richter et al,^30^	349	advanced systolic HF	Austria	75	4.9	195	ELISA	6
Velders et al,^31^	5385	STEMI treated with PPCI (PLATO trial)	Multicenter	59	1	199	ECLIA	5
Dallmeier et al,^32^	1029	Stable CHD	Germany	59	10	162	ECLIA	6
Wallentin et al,^33^	14 798	Atrial fibrillation	Multicenter	70	1.9	1061	ECLIA	6
Eggers et al,^34^	453	Acute chest pain	Germany	66	5.8	92	RIA	7
Dieplinger et al,^35^	530	ICU patients	Austria	68	0.25	118	ECLIA	4
Tzikas et al,^36^ Skau et al,^37^	1804 847	Acute chest pain AMI	Germany Sweden	62 70	0.5 6.9	63 207	Other Other	6 7

Abbreviations: AMI, acute myocardial infarction; CAD, coronary artery disease; CHD, coronary heart disease; CRT, cardiac resynchronization therapy; DEAL‐HF, Deventer‐Alkmaar Heart Failure study; DHS, Dallas Heart Study; ECLIA, electrochemiluminescence assay; ELISA: enzyme‐linked immunosorbent assay; FHS, Framingham Heart Study; FRISC II: Fragming and Fast Revascularization during Instability in Coronary artery disease II; GUSTO‐IV trial: Global Utilization of Strategies to Open Occluded Arteries (GUSTO)‐IV trial; ICTUS: Invasive vs Conservative Treatment in Unstable coronary Syndromes; ICU, intensive care unit; LIPSIA‐N‐ACC: Leipzig Immediate Percutaneous coronary Intervention Acute myocardial infarction N‐Acetyl Cysteine; NSTE‐ACS: Non‐ST‐elevation myocardial infarction; PIVUS: Prospective Investigation of the Vasculature in Uppsala Seniors; RBS: Rancho Bernardo Study; RIA: radioimmunoassay; STEMI: ST‐elevation myocardial infarction; ULSAM: Uppsala Longitudinal Study of Adult Men; al‐HeFT, Valsartan Heart Failure Trial.

**Table 2:** Estimates of association between GDF‐15 levels and risk of adverse outcomes included in the meta‐analysis

Study	Endpoints	Comparison	HR (95% CI)	Adjustments
Wollert et al,^11^	All‐cause mortality	Per SD	1.49 (1.2‐1.85)	Age, gender, delay time, current smoking, history of HTN, hypercholesterolemia, diabetes, previous angina pectoris, MI, revascularization, history of HF, and ST‐segment depression≥0.5 mm
Eitel et al,^12^	Mortality	Per SD	2.51 (1.59‐3.96)	Unadjusted
	MACE^a^	Per SD	2.51 (1.58‐3.96)	
Kempf et al,^13^	Mortality	Per SD	1.55 (1.14‐2.11)	Age, gender, delay time, current smoking, hypertension, diabetes mellitus, history of myocardial infarction, and trial (ASSENT‐2 vs ASSENT‐plus)
Eggers et al,^14^	Death	Per log‐unit	3.4 (2.0‐5.8)	Age, gender, diabetes, heart failure, and previous MI
	Death/recurrent MI		1.9 (1.3‐2.8)	
Kempf et al,^15^	Coronary heart disease mortality	Per SD	2.4 (1.7‐3.4)^b^	Age, gender, HTN, diabetes, smoking, LDL/HDL‐ratio, number of diseased vessels, history of MI, and all indicated biomarkers
			1.6 (1.2‐2.1)^c^	
Khan et al 16	Death	Per log‐unit	1.83 (1.06‐3.15)	Age, gender, previous history of AMI, HF, HTN, DM, smoking history, territory of infarction, STEMI or NSTEMI, Killip class, eGFR, troponin I, therapy with ACE inhibitors, angiotensin receptor blockers and beta‐blockers, NT‐proBNP, and GDF‐15
	Death or heart failure		1.77 (1.03‐3.05)	
Damman et al,^17^	Death	Highest level vs lowest t (>1800 ng/L vs <1200 ng/L)	6.12 (3.45‐10.9)	Unadjusted
	Death or spontaneous MI		3.14 (2.18‐4.52)	
Lin et al,^18^	Death or HF	Per log‐unit	13.39 (2.8‐63.89)	Age, DM
Schopfer et al,^6^	All‐cause mortality	Highest tertile vs lowest	2.73 (1.80‐4.15)	Age, gender, race, smoking, HTN, DM, eGFR, stroke, LDL, exercise capacity, inducible ischemia, NT‐proBNP, CRP, leptin
	MI, stroke, or CV death		1.59 (0.99‐2.55)	
Bonaca et al, 2011^19^	Death	Highest level vs lowest *t* (>1800 ng/L vs <1200 ng/L)	1.91 (0.84‐4.32)	Age, sex, BMI, DM, HTN, current smoking, prior MI, qualifying event, and creatinine clearance, BNP, hsCRP
		Per log‐unit	2.95(1.65‐5.26)	
	Death/MI	Highest level vs lowest *t* (>1800 ng/L vs <1200 ng/L)	1.52 (1.05‐2.19)	
		Per log‐unit	2.14(1.58‐2.91)	
Izumiya et al,^7^	All‐cause mortality/cardiac events*	Per log‐unit	4.74 (1.26‐17.88)	Age, atrial fibrillation, BNP
Wang et al,^9^	Death	Highest quartile vs lowest	3.7 (2.34‐5.86)	Age, sex, BMI, SBP, HTN therapy, diabetes, cigarette smoking, total cholesterol, HDL cholesterol
		Per SD	1.66 (1.51‐1.81)	
	Major cardiovascular event	Highest quartile vs lowest	1.56 (1.03‐2.36)	
		Per SD	1.26 (1.12‐1.41)	
Daniels et al,^20^	Coronary revascularization, MI or CVD death	Highest quartile vs lowest	1.59 (0.96‐2.64)	Age, sex, DM, HTN, current smoking, SBP, total cholesterol, HDL cholesterol, creatinine clearance, BMI
	CVD death		2.46 (1.17‐5.18)	
	All–cause death		2.56 (1.66‐3.94)	
Eggers et al,^21^	All‐cause mortality	Per log‐unit	4.0 (2.7‐6.0)	Sex, HTN, diabetes, HDL cholesterol, LDL cholesterol, current smoking, BMI, previous CVD, ln (CRP), and ln (eGFR)
	CVD mortality		2.3 (1.1‐5.0)	
Rohatgi et al,^22^	All‐cause mortality	Highest level vs lowest (>1800 ng/L vs <1200 ng/L)	3.5 (2.1‐5.9)	Age, sex, race, HTN, diabetes, current smoking, hypercholesterolemia, low HDL‐cholesterol, BMI, CKD stage, LV mass/body surface area, and history of CVD
		Per log‐unit	2.4 (1.7–3.4)	
	CV death	Highest level vs lowest(>1800 ng/L vs <1200 ng/L)	2.5 (1.1‐5.8)	
		Per log‐unit	1.8 (1.1‐3.2)	
Wallentin et al,^23^	All‐cause mortality	Per SD	1.35 (1.18‐1.53)	Age, current smoking, BMI, systolic blood pressure, antihypertensive treatment, total cholesterol, HDL cholesterol, lipid‐lowering treatment, type 2 diabetes, previous cancer, troponin T, NT‐proBNP, Cystatin C, CRP
	CVD mortality		1.22 (1.01‐1.48)	
Lok et al,^24^	All‐cause mortality	Per SD	1.41 (1.11‐1.78)^d^	Age, gender, eGFR HF etiology NT‐proBNP GDF‐15, hs‐TnT, Gal‐3 and/or hs‐CRP
Foley et al,^25^	All‐cause mortality	Per log‐unit	5.59 (2.69‐11.4)	Unadjusted
	CVD mortality		5.31 (2.31‐11.9)	
	CVD mortality or heart failure hospitalizations		3.77 (1.75‐7.90)	
Lajer et al,^26^	All‐cause mortality	Highest quartile vs lowest	4.86 (1.37‐17.30)	Sex age, smoking, A1C, systolic BP, cholesterol GFR, NT‐proBNP, antihypertensive treatment, and a history of cardiovascular events at baseline
	CVD mortality		5.59 (1.23‐25.43)	
Schnabel et al,^27^	Non‐fatal MI and CV mortality	Per SD	1.59 (1.25‐2.02)	Age, sex, BMI, LDL/HDL ratio, smoking, diabetes, hypertension, and number of diseased vessels.
Wiklund et al,^28^	All‐cause mortality	Highest level vs lowest (>1800 ng/L vs <1200 ng/L)	2.61 (1.53‐4.45)^e^	Blood draw, BMI, and smoking history
			2.20 (1.47‐3.42)^f^	
Kempf et al,^5^	All‐cause mortality	Per log‐unit	2.26 (1.52‐3.37)	Age, male gender, ischemic etiology, NYHA functional class, LVEF, ln NT‐proBNP, ln creatinine, Hb, ln uric acid, ln GF‐15.
Widera et al,^29^	Death	Per SD	2.4 (1.9‐3.0)^g^	Unadjusted
Richter et al,^30^	All‐cause mortality	Per SD	1.22 (1.03‐1.45)	Age, sTRAIL, NT‐proBNP, sFAS, GDF‐15, Fractalkine, GDF‐15, COPD
Velders et al,^31^	CVD mortality	Highest quartile vs lowest	2.27 (1.32‐4.09)	Age, gender, DM, Killip class, admission heart rate, admission SBP, history of congestive heart failure, peripheral arterial disease, cystatin C, previous MI, previous PCI, previous CABG, randomized treatment arm (ticagrelor/clopidogrel), extent of CAD, NT‐proBNP, cTnT‐hs
		Per SD	1.42 (1.25‐1.61)	
Dallmeier et al,^32^	All‐cause mortality	Highest level vs lowest (>1800 ng/L vs <1200 ng/L)	1.73 (1.02‐2.94)	Age, sex, BMI, smoking, diabetes, HTN, TC, HDL‐C, use of statins, cystatin C, NT‐proBNP, hs‐CRP, and hs‐cTnT
Wallentin et al,^33^	All‐cause mortality	Highest quartile vs lowest	2.10 (1.63‐2.73)	Randomized treatment, previous warfarin/vitamin K antagonist treatment, geographic region, age, sex, BMI, smoking status, sBP, heart rate, atrial fibrillation type, DM, history of symptomatic congestive HF, previous stroke/systemic embolism/transient ischemic attack, HTN, previous MI, previous peripheral artery disease/coronary artery bypass graft/percutaneous coronary intervention, cTnI, NT‐proBNP, cystatin‐C.
Eggers et al,^34^	All‐cause mortality	Per SD	2.0 (1.6‐2.5)	Age, hypertension, diabetes, previous AMI, previous heart failure, heart rate and sBP on admission, ST‐segment depression on admission, and peak cTnI N0.07 μg/L (within 24 hours), GRACE risk score.
Dieplinger et al,^35^	All‐cause mortality	Highest level vs lowest (>3470 ng/L vs < ng/L)	4.83 (3.05‐7.64)	Unadjusted
		Per SD	2.06 (1.72‐2.46)	
Tzikas et al,^36^	Death/MI	Per SD	1.57 (1.13‐2.19)	GRACE score variables: heart rate, (log) creatinine, ST changes in ECG, age, systolic blood, pressure and Killip class.
Skau et al,^37^	All‐cause mortality	Per log‐unit	2.57(2.31‐2.85)	Unadjusted

ACE, angiotensin converting enzyme; AMI, acute myocardial infarction; BMI, body mass index; BNP, pro‐brain natriuretic peptide; CKD, chronic kidney disease; CI, confidence interval; CRP, C‐reactive protein; cTnI, cardiac troponin I; CVD, cardiovascular disease; DM, diabetes mellitus; ECG, electrocardiography; eGFR, estimated glomerular filtration rate; GDF‐15, growth differentiation factor‐15; HDL, high‐density lipoprotein; HF, heart failure; HR, hazard ratio; hsCRP, high sensitivity C‐reactive protein; HTN, hypertension; LDL, low‐density lipoprotein; LVEF, left ventricular ejection fraction; MACE, major adverse cardiac events; MI, myocardial infarction; NYHA, New York Heart Association; NT‐proBNP, N‐terminal pro‐brain natriuretic peptide; NSTEMI, non‐segment elevation myocardial infarction; STEMI, segment elevation myocardial infarction; SBP, systolic blood pressure.

a MACE: death, reinfarction, and new congestive heart failure within 6 months after the index event.

b Stable angina group.

c ACS group.

d Based on 451 patients with diabetic nephropathy.

e Male cohort.

f Twin cohort.

g Derivation cohort (n = 754).

### 3.3 GDF‐15 and the risk of cardiovascular mortality

**Figure 2:**
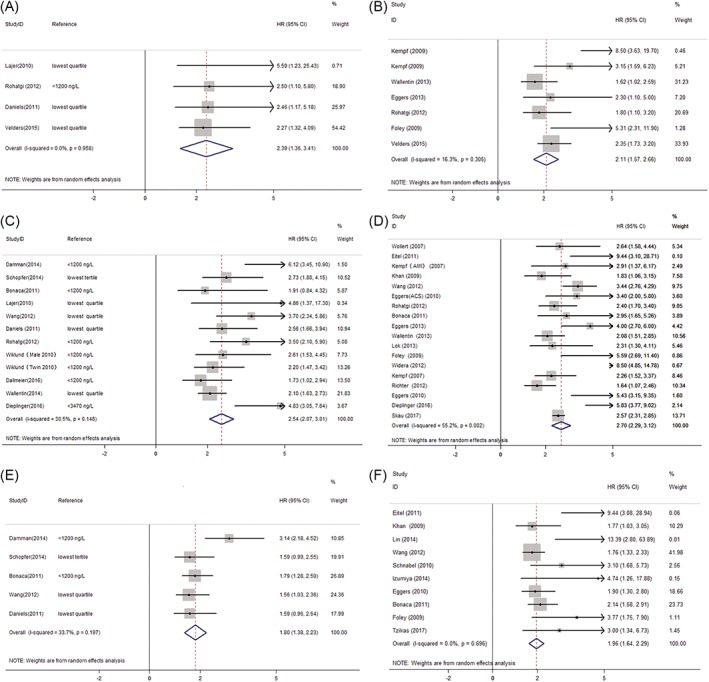
A, Forest plot showing the association between growth differentiation factor‐15 (GDF‐15) levels and cardiovascular mortality (categorical variable). Solid squares indicate HR in each study, and the size of the square is proportional to the precision of HR. The 95% CI are denoted by lines and empty diamonds represent pooled HR. B, Forest plot showing the association between GDF‐15 levels and cardiovascular mortality (continuous variable). Solid squares indicate HR in each study, and the size of the square is proportional to the precision of HR. The 95% CI are denoted by lines and empty diamonds represent pooled HR. CI, confidence interval; HR, hazard ratio. C, Forest plot showing the association between GDF‐15 levels and all‐cause mortality (categorical variable). Solid squares indicate HR in each study, and the size of the square is proportional to the precision of HR. The 95% CI are denoted by lines and empty diamonds represent pooled HR. CI, confidence interval; HR, hazard ratio. D, Forest plot showing the association between GDF‐15 levels and all‐cause mortality (continuous variable). Solid squares indicate HR in each study, and the size of the square is proportional to the precision of HR. The 95% CI are denoted by lines and empty diamonds represent pooled HR. E, Forest plot shows the association between GDF‐15 levels and complex adverse outcome (categorical variable). Solid squares indicate HR in each study, and the size of the square is proportional to the precision of HR. The 95% CI are denoted by lines and empty diamonds represent pooled HR. F, Forest plot showing the association between GDF‐15 levels and complex adverse outcome (continuous variable). Solid squares indicate HR in each study, and the size of the square is proportional to the precision of HR. The 95% CI are denoted by lines and empty diamonds represent pooled HR. ACS, acute coronary syndrome; AMI, acute myocardial infarction; CI, confidence interval; HR, hazard ratio

Eight studies reported cardiovascular mortality as outcomes. Four studies handling the data as a categorical variable demonstrated that the pooled HR for highest GDF‐15 category vs lowest was 2.39 (95% CI, 1.36‐3.41) (Figure 2A). Moreover, when regarded GDF‐15 level as a continuous variable, the pooled HR for cardiovascular mortality from six studies was 2.11 (95% CI, 1.57‐2.66) per log‐unit ng/L increment using a random effect model. (Figure 2B).

### 3.4 GDF‐15 and the risk of all‐cause mortality

Figure 2C showed the pooled HR for all‐cause mortality comparing the highest GDF‐15 category with lowest of GDF‐15 level (HR, 2.54; 95% CI, 2.07‐3.01). When GDF‐15 concentration increased one log‐unit ng/L, 2.7‐fold of the risk of all‐cause mortality correspondingly varied (HR, 2.70; 95% CI, 2.29‐3.12 (Figure 2D).

### 3.5 GDF‐15 and the risk of complex adverse outcome

We observed that GDF‐15 concentration was associated with an increased risk of complex adverse outcome (HR, 1.80; 95% CI, 1.38‐2.23), when pooling data from six studies reporting the estimates as categorical variables. (Figure 2E) Every log‐unit ng/L increase in GDF‐15 concentration was associated with a 95% increase in the risk of complex adverse outcome from pooling the nine studies reporting estimates risk as continuous variables (HR, 1.96; 95% CI, 1.64‐2.29). (Figure 2F).

### 3.6 Heterogeneity measurement

**Figure 3:**
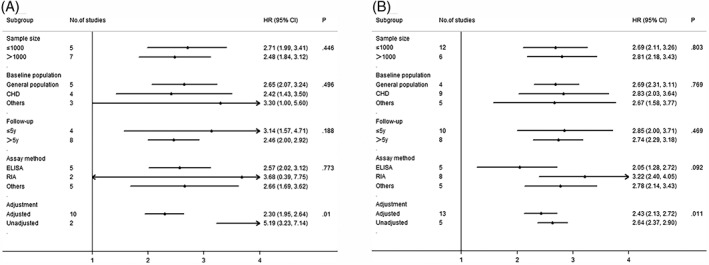
A, Pooled HR and 95% CI by subgroups (sample size, baseline population, follow‐up, and assay method) of the association between growth differentiation factor‐15 (GDF‐15) levels and all‐cause mortality (categorical variable). Black dots represent HRs and bars indicate 95% CIs. B, Pooled HR and 95% CI by subgroups (sample size, baseline population, follow‐up, assay method) of the association between GDF‐15 levels and all‐cause mortality (continuous variable). Black dots represent HRs and bars indicate 95% CIs. CI, confidence interval; HR, hazard ratio; CHD, coronary heart disease; ELISA, enzyme‐linked immunosorbent assay; RIA, radioimmunoassay

As the heterogeneity inspection tools, meta‐regression, analysis and sensitivity analysis were carried out aimed at exploring the potential sources of heterogeneity on all‐cause mortality regarding GDF‐15 as a categorical variable or continuous variable in our study. The detailed results were displayed in the Figure 3A, B. Meta‐regression analysis was executed to exploit the heterogeneity stratified by such feasible causes, sample size, baseline population duration of follow‐up, assay method, and adjustment. In evidence, the aforementioned four factors were unable to strike significant heterogeneity, but whether to adjust the confounding variables may be one of the sources of heterogeneity. We conducted a sensitivity analysis based on all‐cause mortality. In the sensitivity analyses, the results suggested that the pooled HRs or 95% CI did not reflect significant difference when omitting one of the studies from the analysis. (Figure S1 and S2, Supporting Information).

### 3.7 Publication bias

Egger test showed that no publication bias was observed (coefficient 1.772, *P* = 0.201, 95% CI: −1.15‐4.66) when considered as categorical variable. The similar results also identified the relationship as the continuous variable (coefficient 1.214, *P* = 0.121, 95% CI, −0.36‐2.78). (Figure S3 and S4).

## 4 DISCUSSION

CVD, one of the major causes of death, has drawn comprehensive attention worldwide. Serum biomarkers play a crucial role in diagnosing CVDs, moreover, providing the predictive value on adverse outcomes. Apart from traditional cardiac markers, some newly discovered serum biomarkers have been found from large amounts of research. The role of GDF‐15 as a potential risk predictor in CVDs has been investigated popularly in recent years. To the best of our knowledge, it is probably the first attempt to analyze the data from the relative literature to evaluate the association on the risk of CVDs or all‐cause mortality.

Two studies were conducted on meta‐analysis of the relationship between GDF‐15 and the different prognosis of acute coronary syndromes and heart failure separately. One of them, GDF‐15 is classified as a categorical variable for statistical analysis,^38^ and the other is as a continuous variable.^39^ In our meta‐analysis, 31 studies were included for the further integration analysis comprehensively. It was calculated that GDF‐15 was significantly increased when adverse cardiovascular events occur (cardiovascular mortality, all‐cause mortality, and complex adverse outcome). Meanwhile, GDF‐15 was classified into categorical and continuous data for analysis, respectively. Thus, we can more clearly and intuitively infer the relationship between GDF‐15 and the different outcome of CVD or all‐cause mortality. Our meta‐analyses summarize the results of different cohort studies and evaluate the association between GDF‐15 levels and the risk of CVDs or all‐cause mortality. It is indicated that higher GDF‐15 levels have an adverse relationship on the risk of CVDs or all‐cause mortality. In view of our results, compared with the lowest GDF‐15 levels, those with the highest levels have a 139% increment in the risk of CVD mortality, 154% increment in the risk of all‐cause mortality, and 80% increment in the risk of composite of death or cardiac events. When GDF‐15 level was calculated as a continuous variable, there is a 111% increment in the risk of CVD mortality per log‐unit increment, 170% increment in the risk of all‐cause mortality per log‐unit increment and 96% increment in the risk of composite of death or cardiac events. So, qualitatively and quantitatively assessment for the association between GDF‐15 levels and adverse outcomes has been performed respectively to verify the hypothesis.

Plenty of clinical researches had demonstrated that GDF‐15 is independently related to a variety of CVDs. Moreover, growth differentiation factor 15 provided more prognostic information in assessing the prognosis for the risk of adverse cardiac events or all‐cause mortality. The majority of the included literature in this analysis had verified conclusions drawn from a large number GDF‐15 prospective clinical trials, indicating that GDF‐15 may play a protective role in the pathophysiological processes.

In this meta‐analysis, we have fully integrated the studies on the basis of different research groups, including general population, cardiac disease patients, and various of diseases cases. Numerous studies have demonstrated that GDF‐15 engaged in predicting the prognosis of the diseases of different baseline populations. HF, as the end‐stage of a variety of heart disease, earlier diagnosis appears to be particularly crucial. Studies of research population of HF has showed that the concentration of GDF‐15 was markedly elevated, moreover, providing prognostic information with clinical value on all‐cause mortality.^5^ It was proved by experiment that GDF‐15 plays a protective role by inhibiting apoptosis, hypertrophy, and adverse remodeling in the injured heart. GDF‐15 also participated in the development of cardiac remodeling in HF with a function of counterworking hypertrophy and apoptosis through PI3K‐Akt, ERK1/2, and SMAD2/3 signaling pathways.^2^ A study of a large sample of AF has identified that GDF‐15 is an indicator for the prognosis of major bleeding and death.^33^ Owing to the incidence trend of ischemic heart disease, the application of GDF‐15 on early diagnosis and prognosis of disease is widely studied. In an animal model, it was confirmed that GDF‐15 played a cardioprotective role. After the occurrence of ischemia‐reperfusion (I/R), the expression of GDF‐15 was sharply enhanced in the cardiomyocytes as an endogenous protective cytokine against I/R‐induced cardiomyocyte apoptosis, possibly through PI3K‐Akt‐dependent signaling pathways. In addition, it seemed to be mediated by the approach of induction of nitric oxide (NO) synthase‐2, production of NO and peroxynitrite formation. Meanwhile, some relevant pro‐inflammatory cytokines (interferon‐γ and interleukin‐1β) involved in the induction of GDF‐15 through NO‐dependent pathways.^3^ Besides, GDF‐15, has provided more valuable information for risk stratification and prognosis in CVDs.^11^ An epidemiological research demonstrates that GDF‐15 levels are associated with carotid artery intima‐media thickness, plaque burden, and endothelial dysfunction in elderly individuals, which may provide insight into disparate mechanism of GDF‐15 pathophysiology.^40^ Some studies included in our meta‐analysis demonstrate that GDF‐15 has performed well both in identifying stable and unstable CAD and in evaluating the prognosis, and likewise, independent of traditional clinical risk biomarkers, such as troponin T, NT‐proBNP, and hs‐CRP.^6^, ^13^, ^15^ To find the potential heterogeneity, we did meta‐regression analysis and sensitivity analysis to identify conceivable sources of the discrepancy from the studies that reported all‐cause mortality as endpoints. Several probable aspects were filtered for the subgroup analyses. Paradoxically, the results of meta‐regression analysis and sensitive analysis was failed to elucidate that where the heterogeneity stems from genuinely. It was inexplicit that which was the initiator of the difference. Furthermore, the results of the sensitivity analysis also indicated that the elimination of any study did not make a significant alteration in the pooled HR.

In conclusion, it can be noted that the present study has stated the association between GDF‐15 and adverse prognosis of CVDs and all‐cause mortality. A large number of research need to be done until GDF‐15 could contribute to clinical and bring more valuable information for clinician.

## 5 LIMITATION

We delimit the studies published in the English language as one of the eligible criteria, which may be a limiting factor. First based on prospective study data, the result of our meta‐analysis is subject to potential bias. Although potential confounders, such as age, sex, and body mass index have been adjusted in most studies, residual confounding cannot be precluded. Second, some studies of small sample size resulting in extremely strong associations may have an impact on pooled estimates. Third, the characteristics of baseline participants differ across included studies which cause the initial production of heterogeneity. Fourth, on account of disparate adjusted variables of each study it is arduous to unify the diverse adjustments for all studies. Fifth, a dose‐effect relationship between the GDF‐15 level and all‐cause mortality or adverse cardiac events is failed to be conducted owing to the lack of indispensable data. In addition, the indefinite cutoff point of GDF‐15 and the various assay methods may influence the results. The relative small sample sizes of studies attenuate the strength of this meta‐analysis. Furthermore, larger numbers of studies are expected to strengthen our results, which may provide more vital information on the diagnosis and prognosis for CVDs.

## 6 CONCLUSION

Elevated GDF‐15 may increase risk of all‐cause mortality and adverse cardiovascular events. Further and more detailed clinical investigations are needed to conduct to identify whether measurement of GDF‐15 can be applied to clinical patients for an effective earlier diagnosis, risk stratification, and prognosis assessment.

### Author contributions

Conceived and designed the experiments: L. Lu Retrieved literature: (S. Xie and L. Liu). Analyzed the data: (S. Xie). Contributed to data analyses: (L. Liu). Wrote the manuscript: S. Xie.

## Supporting information


**FIGURE S1** HR and 95% CI by omitting each study from the eligible studies of the association between GDF‐15 levels and all‐cause mortality (categorical variable). Empty dots represent HRs and bars indicate 95% CIs. CI, confidence interval; HR, hazard ratio

**FIGURE S2** HR and 95% CI by omitting each study from the eligible studies of the association between GDF‐15 levels and all‐cause mortality (continuous variable). Empty dots represent HRs and bars indicate 95% CIs. CI, confidence interval; HR, hazard ratio; ACS: acute coronary syndrome; AMI: acute myocardial infarction

**FIGURE S3** Egger's funnel plot (with pseudo 95% CIs) to detect any publication bias for the association between GDF‐15 level and all‐cause mortality as categorical variables. Empty dots denote effect size of each study; CI, confidence interval

**FIGURE S4** Egger's funnel plot (with pseudo 95% CIs) to detect any publication bias for the association between GDF‐15 level and all‐cause mortality as continuous variables. Empty dots denote effect size of each study; CI, confidence interval
