# Supplementary figures and images for: Growth differentiation factor‐15 and the risk of cardiovascular diseases and all‐cause mortality: A meta‐analysis of prospective studies
Source: Clin Cardiol. 2019 Mar 26;42(5):513–23. doi: 10.1002/clc.23159 (PMC6523003; doi:10.1002/clc.23159)

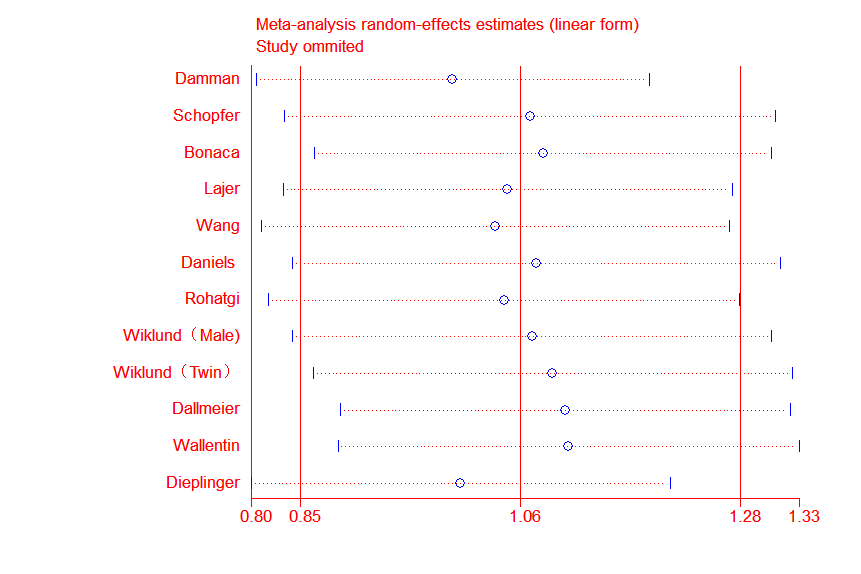

Supplement: Supplementary file 1 — FIGURE S1 HR and 95% CI by omitting each study from the eligible studies of the association between GDF‐15 levels and all‐cause mortality (categorical variable). Empty dots represent HRs and bars indicate 95% CIs. CI, confidence interval; HR, hazard ratio [file CLC-42-513-s001.tif]

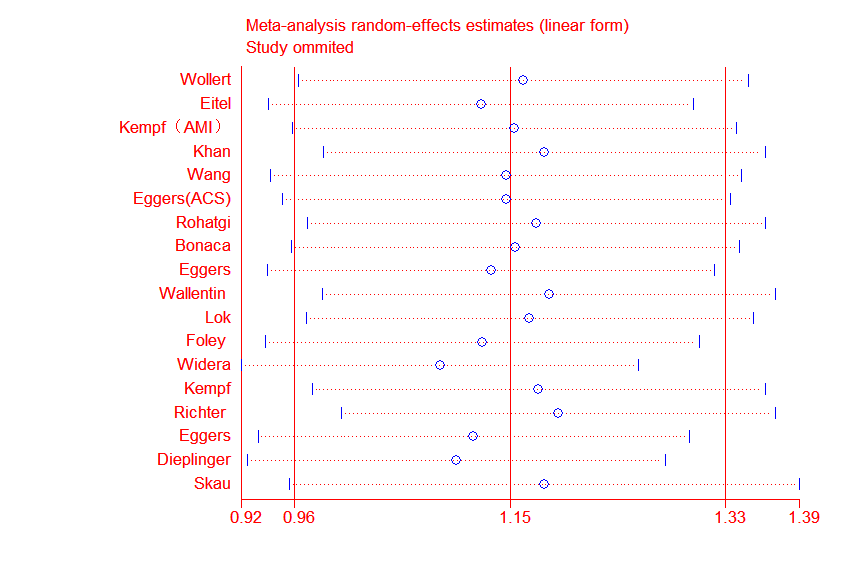

Supplement: Supplementary file 2 — FIGURE S2 HR and 95% CI by omitting each study from the eligible studies of the association between GDF‐15 levels and all‐cause mortality (continuous variable). Empty dots represent HRs and bars indicate 95% CIs. CI, confidence interval; HR, hazard ratio; ACS: acute coronary syndrome; AMI: acute myocardial infarction [file CLC-42-513-s002.tif]

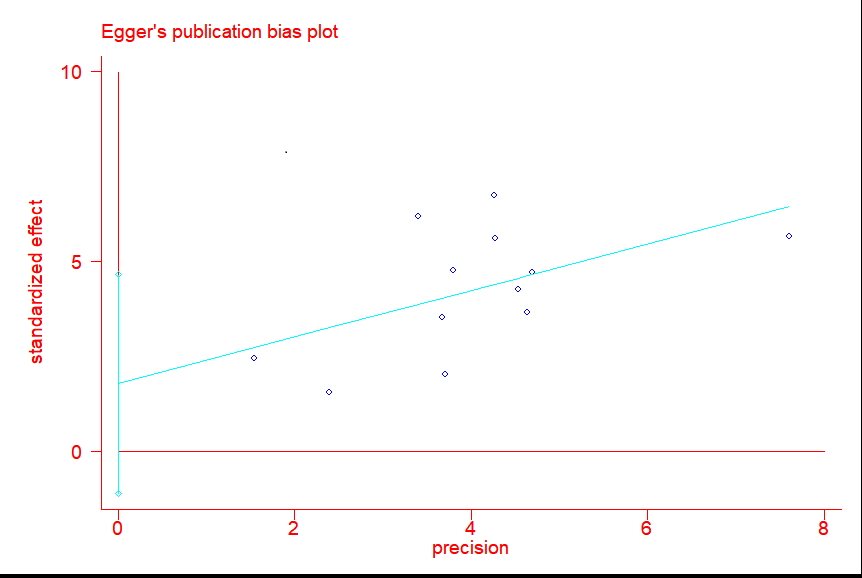

Supplement: Supplementary file 3 — FIGURE S3 Egger's funnel plot (with pseudo 95% CIs) to detect any publication bias for the association between GDF‐15 level and all‐cause mortality as categorical variables. Empty dots denote effect size of each study; CI, confidence interval [file CLC-42-513-s004.tif]

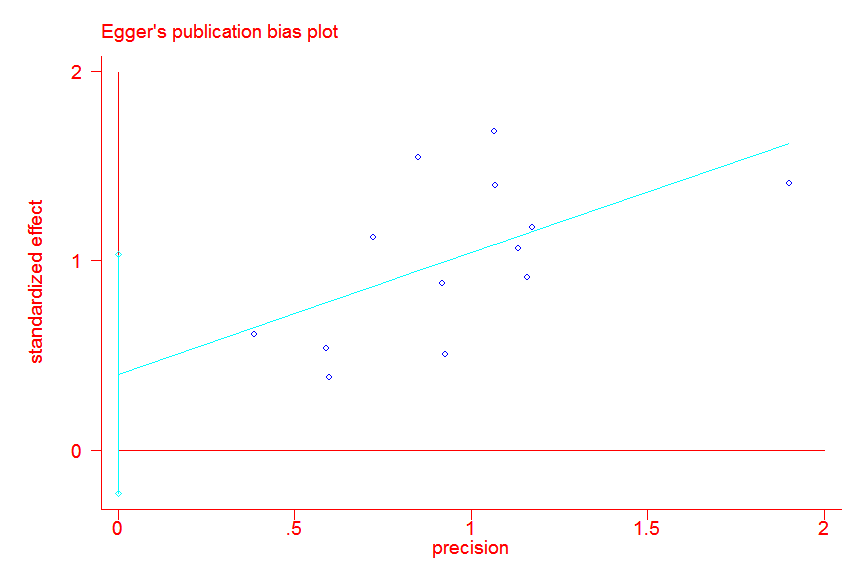

Supplement: Supplementary file 4 — FIGURE S4 Egger's funnel plot (with pseudo 95% CIs) to detect any publication bias for the association between GDF‐15 level and all‐cause mortality as continuous variables. Empty dots denote effect size of each study; CI, confidence interval [file CLC-42-513-s003.tif]
